# Neonatal Abdominal Hemangiomatosis: Propranolol beyond Infantile Hemangioma

**DOI:** 10.1155/2016/9803975

**Published:** 2016-03-27

**Authors:** Siu Ying Angel Nip, Kam Lun Hon, Wing Kwan Alex Leung, Alexander K. C. Leung, Paul C. L. Choi

**Affiliations:** ^1^Department of Paediatrics, The Chinese University of Hong Kong, Prince of Wales Hospital, Shatin, Hong Kong; ^2^Department of Paediatrics, University of Calgary, 200-233 16th Avenue NW, Calgary, AB, Canada T2M 0H5; ^3^Department of Anatomical & Cellular Pathology, Prince of Wales Hospital, Shatin, Hong Kong

## Abstract

Hemangioma is the most common vascular tumor of infancy; presentation is often as cutaneous infantile hemangioma (IH). Cutaneous hemangioma is a clinical diagnosis. Most IHs follow a benign course, with complete involution without treatment in the majority of cases. Visceral hemangioma often involves the liver and manifests as a life-threatening disorder. Hepatic hemangiomas may be associated with high output cardiac failure, coagulopathy, and hepatomegaly which generally develop between 1 and 16 weeks of age. Mortality has been reportedly high without treatment. We report a rare case of a male infant with neonatal hemangiomatosis with diffuse peritoneal involvement, which mimicked a malignant-looking tumor on imaging, and discuss therapeutic options and efficacy. Propranolol is efficacious for IH but generally not useful for other forms of vascular hemangiomas, tumors, and malformations. In our case of neonatal peritoneal hemangiomatosis, propranolol appears to have halted the growth and possibly expedite the involution of the hemangiomatosis without other treatments.

## 1. Introduction

Hemangiomas are the most common vascular tumors of infancy [1–5]. When limited to skin, multiple lesions generally have a benign course and excellent prognosis. In cases of visceral involvement, the morbidity and mortality rates are high [6]. We report a rare case of a male infant with neonatal hemangiomatosis with diffuse peritoneal involvement, which mimicked a malignant-looking tumor on imaging, and discuss therapeutic options and efficacy of treatment of this condition.

## 2. Case Report

**Figure 1:**
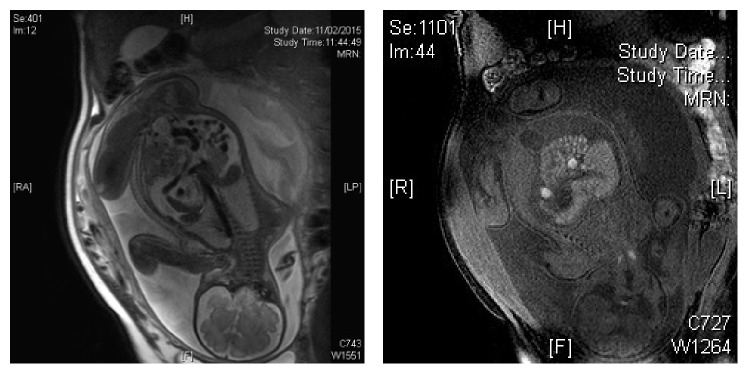
Fetal pelvic MRI showed T1 and T2 isointense soft tissue mass.

A term baby boy, birth weight 4.17 kg, with fetal ascites and abdominal distension was delivered by Cesarean section. Antenatal scan at 20 weeks' gestation showed fetal ascites with normal morphology. Amniocentesis was performed and karyotyping was normal. Viral studies including cytomegalovirus and toxoplasmosis were unyielding. Repeated antenatal ultrasonography scan at 23 weeks showed increased ascites with peritoneal thickness. There was an irregular solid mass arising from left posterolateral peritoneal surface and protruding into the peritoneal cavity. Fetal MRI showed a T1 and T2 isointense soft tissue mass at the left lower quadrant of the abdomen, without cystic or fatty components (Figure 1). The mass extended from the left lower kidney superiorly to left iliac fossa. The soft tissue mass was identified separately from the visceral organs, including the left kidney, and small and large bowel loops. The mass was, however, inseparable from the left lateral peritoneal lining. There was a large amount of ascites. Follow-up ultrasound scan at 35 weeks showed increasing tumor size with multiple peritoneal deposits at subdiaphragmatic surfaces. Overall features were suspicious of a malignant soft tissue tumor with peritoneal origin with metastases. The differential diagnoses included fibrosarcoma, rhabdosarcoma, and peritoneal neuroblastoma.

**Figure 2:**
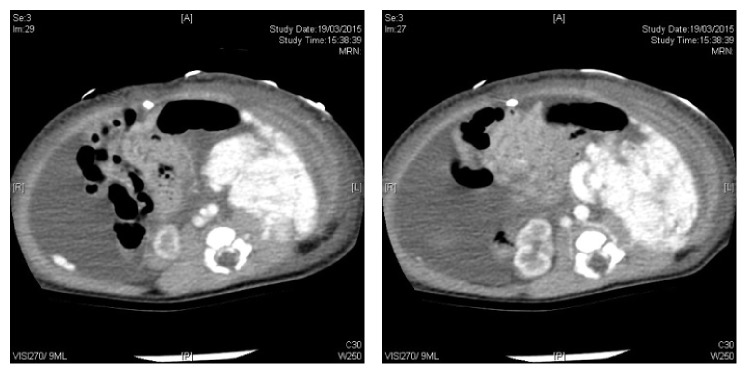
Postnatal CT scan showed a large multilobulated enhancing mass.

**Figure 3:**
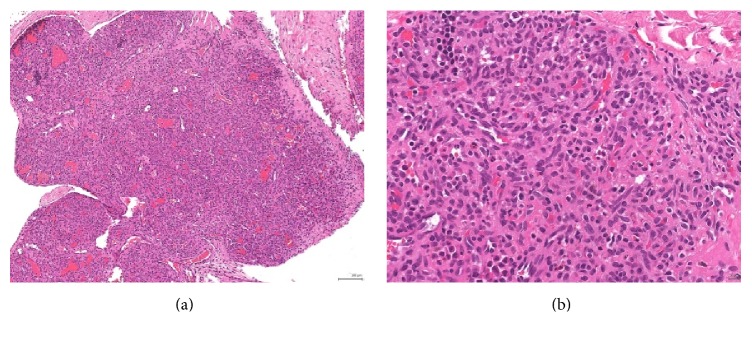
(a) Lobulated proliferation of small irregular capillary-sized vascular channels. (b) The lining endothelial cells are bland looking with no cytological atypia.

**Figure 4:**
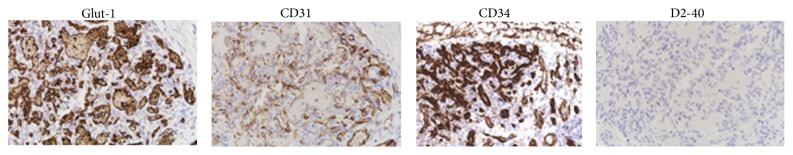
The endothelial cells are positive for Glut-1 which favors hemangioma rather than vascular malformation. They are also positive for CD31 and CD34 (vascular markers). They are negative for D2-40 stain which rules out lymphangioma. The overall feature is hemangioma.

The infant was born at term with gross abdominal distension and respiratory distress, requiring mechanical ventilation support at birth. He was successfully extubated on day 2 of life. Postnatal CT scan revealed a large multilobulated enhancing mass, 5.8 × 4.3 × 8.6 cm in size, along the left paracolic gutter, extending down to the left iliac fossa. A large tortuous vein was noted to be draining directly into the inferior vena cava and there were multiple small (up to 1.6 cm) avidly enhancing lesions present in the subphrenic and perihepatic regions. They were compatible with peritoneal metastases on contrast imaging (Figure 2). The large and small bowels were all displaced. There was no lymph nodes involvement. Overall features were suggestive of a malignant soft tissue tumor with peritoneal deposits. Tumor markers including alpha fetoprotein, HCG, and urine catecholamine were unremarkable. Ultrasound-guided paracentesis yielded chylous exudate but no malignant cells. The infant was put on medium-chain oil-based milk formula. Laparoscopic biopsy was performed under general anesthesia on day 13 to obtain tissue for histological diagnosis. The infant tolerated the procedure without any complication. There were multiple vascular tumors with largest bulk at the left retroperitoneal region and multiple peritoneal deposits of similar nature scattered throughout the abdomen. Immunohistochemical staining of tumor cells was positive for Glut-1, CD31, and CD34 and negative for pan-cytokeratin (AE1/AE3), epithelial membrane antigen (EMA), desmin, myogenin, S100, c-kit, and D2-40 (Figures 3 and 4). These immunofluorescence tests implied endothelial vascular lesions. The overall feature is hemangioma and not vascular malformation or lymphangioma. The morphology was compatible with benign lobulated capillary hemangioma of peritoneum with angiomatosis. There was no cutaneous involvement and blood cell counts were not affected.

Echocardiogram and ultrasound of the brain were unremarkable. The infant was treated with propranolol at 2 mg/kg/day in three divided doses. Clinically the infant continued to do well and had normal growth and development. He tolerated propranolol well. There was clinically no abdominal distension or palpable abdominal mass. Four months after initial presentation, repeat ultrasound of the abdomen showed a reduction in size of the soft tissue and decrease in numbers of small peritoneal deposits. Latest ultrasound scan at 8 months showed the heterogeneous left flank hemangioma measuring 2.4 × 0.9 cm.

## 3. Discussion

This is an unusual case of hemangiomatosis involving the visceral omentum. Infantile hemangioma is the most common vascular tumor of infancy, with an incidence between 1 and 12%, varying by race [2, 3]. Female infants are 4 times more likely to be affected. There are various presentations of hemangiomas in infancy, most commonly as cutaneous manifestations. Cutaneous hemangioma is a clinical diagnosis. Most cutaneous lesions follow a benign course [6]. In approximately 10–20% of patients, the lesions are multiple. Fifty percent of the lesions completely involute without treatment by age 5, 75% by age 7, and nearly 90% by age 9. Visceral hemangioma, on the other hand, can be a severe and life-threatening disorder [7, 8]. Liver is the most commonly involved internal organ, followed by lungs, brain, and intestine. Hepatic hemangiomas may be associated with high output cardiac failure, coagulopathy, and hepatomegaly which generally develop between 1 and 16 weeks of age. Mortality has been reported to be as high as 81% without treatment and reduced to 29% with treatment [3].

Imaging is useful for visceral hemangiomas. There are typical radiological features which can help with the diagnosis, such as vascular pooling of contrast within the tumor and a centripetal enhancement pattern on dynamic scan. However, in our reported case, the lack of radiological features in combination with the malignant appearance and rapid growing nature warrants a histological diagnosis to exclude other primary neoplasms.

Most cutaneous hemangiomas do not require treatment and only warrant close observation. Indications for active intervention include severe or recurrent hemorrhage unresponsive to treatment, threatening ulceration in areas where serious complications might ensue, interference with vital structures, pedunculated hemangiomas, and significant disfigurement. In recent years, propranolol, a nonselective *β*-blocker, has been used as a first-line treatment of problematic infantile hemangioma. Multiple, small retrospective case series confirm the effect of propranolol [9]. A 2013 meta-analysis of 35 studies demonstrated propranolol's superior efficacy over steroids, vincristine, and laser treatment [10]. It has been proposed that the reduction in size of the tumor results from vasoconstriction due to decreased nitric oxide release, downregulation of vascular growth factors, and induction of apoptosis [11–13]. Our patient also showed significant response to propranolol treatment in terms of size reduction.

In conclusion, we report an unusual case of congenital omental hemangiomatosis which behaves like a malignant-looking neoplasm radiologically. Yet it responded well to propranolol treatment. Propranolol may be considered as first-line therapy for congenital hemangiomas that are extensive and involve the viscera. Life-threatening and complicated cases of hemangiomatosis are challenging conditions especially in the neonates. Other treatment options include corticosteroid, cyclophosphamide, and laser therapy [3, 5, 7, 8, 12].
